# Attention deficit/hyperactivity disorder and risk of injuries: A systematic review and meta-analysis

**DOI:** 10.5249/jivr.v9i2.858

**Published:** 2017-07

**Authors:** Shahrokh Amiri, Homayoun Sadeghi-Bazargani, Soulmaz Nazari, Fatemeh Ranjbar, Salman Abdi

**Affiliations:** ^*a*^Research Center of Psychiatry & Behavioral Sciences, Tabriz University of Medical Sciences, Tabriz, Iran.; ^*b*^Road Traffic Injury Research Center, Department of Statistics & Epidemiology, Tabriz University of Medical Sciences, Tabriz, Iran.; ^*c*^WHO Collaborating Center on Community Safety Promotion, Karolinska Institute, Stockholm, Sweden.

**Keywords:** Attention deficit hyperactivity disorder, Injuries, Accidents, Systematic review

## Abstract

**Background:**

This study systematically reviewed the literature in order to determine the effect of Attention-Deficit Hyperactivity Disorder (ADHD) on injuries and assessed the magnitude of the potential association.

**Methods:**

A systematic review of the studies examining the association of ADHD and injuries was carried out across multiple databases. Odds ratios and standardized mean differences were pooled.

**Results:**

A total of 35 studies were selected for quantitative analysis. The association of ADHD and injuries was confirmed over the meta-analysis of eligible studies. The odds ratio pooled over all comparative studies was 1.96(95% CI: 1.6-2.4) using random effects model. Pooled odds ratio of 2.1 and 2.17 were calculated respectively when cohort and case-control studies or just cohort studies were included. The pooled odds ratio reduced to 1.8(CI:1.45-2.3) when studies on specific injuries were removed. For studies comparing scores of rating scales, the pooled standardized mean difference was 0.61(95% CI: 0.03-1.2).

**Conclusions:**

Those with ADHD are nearly two times more likely to be injured.

## Introduction

Injuries are considered a major public health problem. They are the leading cause of death among young adults in United States and above 28 million injuries occur annually requiring emergency medical care.^1^ Globally about 1.24 million people die on roads and road traffic accidents cause 20-50 million nonfatal inju-ries each year. ^2^ Other types of injuries such as falls and burns lead to a noticeable burden of injuries worldwide leading to mortality and other subsequent outcomes such as disability, psychological and economic consequences.^2-5^ To prevent injuries it is crucial to map out its epi-demiology and distinguish injury risk factors. Psychologi-cal factors have always been a matter of interest in the field of injury research. Attention-deficit hyperactivity disorder (ADHD) is a childhood-onset psychiatric disor-der. However, it is also relatively common among adults, with a prevalence reaching up to 5% in the general population. ^6^

Studies have shown that the injuries are associated with ADHD.^7-10^ Several studies have also been specifically conducted to show an association between ADHD and various types of injuries in childhood. It has been reported that ADHD may be associated with burns, ^11^ fractures,^9,12,13^ dental trauma ^14,15^ and traffic injuries. ^16^ ADHD is a treatable and easily detectable condition and if its potential role in injuries is clearly and trustfully determined, international safety promotion plans can largely benefit from the synthesized evidence. The aim of this study was to systematically review the available literature in order to determine the effect of ADHD on injuries and assess the magnitude of the potential association between unintentional or undescribed injuries and ADHD when assuming ADHD as a risk indicator of injuries.

## Methods

This study is a systematic review of all published peer reviewed articles in the period from 2000 to 2014 that have examined the association between ADHD and injuries regardless of the age group or the injury mechanisms. 

**Exposure and outcome:**

With respect to the aim of the study, the exposure was defined as having ADHD diagnosis of any subtype (DSM-defined ADHD or ICD-defined hyperkinetic disorder, as well as historic variants) or to meet accepted criteria for clinical levels of symptoms on validated ADHD rating scales. The average scores on validated ADHD rating scales were considered the exposure of interest. 

The outcome of interest was being injured through an accidental or undescribed injury mechanism. Various types of injury mechanisms according to the international classification of diseases, such as traffic injuries, burns, falls, occupational injuries etc., were included.

**Eligibility criteria and selection of studies:**

The authors planned to include studies with a controlled design (including cohort and case-control studies) that were published in peer-reviewed journals at any time from 2000 to 2014.

To ensure minimum methodological standards, only the studies published in peer-reviewed journals were included. Moreover, studies with low reported quality of conduction ,assessed by an expert epidemiologist, were excluded from the study. The main items of interest in assessing the quality of published articles and risks of bias included; valid assessments of the exposure ; valid assessments of the outcome; risk of selection bias; risk of information bias; ability of the study to exclude reverse causality detection between ADHD and injuries; statistical validity and quality of reporting. 

 We defined the participants enrolled in various studies to have either a diagnosis of ADHD or to meet the accepted criteria for clinical levels of symptoms on validated ADHD rating scales. We also included the studies comparing the participants according to their screening score on clinical levels of symptoms on validated ADHD rating scales even if the participants were not categorized to have ADHD. Studies in which the enrollment depended on the presence of rare comorbid conditions were excluded. Studies specifically conducted to assess the association of ADHD and brain traumatic injuries were excluded. No age, gender and injury mechanism restriction was applied in this review, however, only the studies written in English were enrolled. Articles were initially screened on the basis of titles and abstracts, while the assessment of articles for final inclusion was based on full text papers assessed independently by two of the authors. Disagreements were all resolved through discussion or joint reexamination of the articles.

**Search Strategy:**

A broad range of electronic databases were searched. OVID SP, PubMed, Medline, Science Direct and EMBASE data sources were searched using MESH terms and free keywords as following

 ("Attention Deficit Disorder with Hyperactivity"[Mesh]) AND ("Wounds and Injuries"[Mesh] OR "injuries" [Subheading] OR "Accidental Falls"[Mesh] OR "Accidents"[Mesh] OR "Burns"[Mesh] OR "Accidents, Traffic"[Mesh] OR "Fractures, Bone"[Mesh])

In free keyword search, the exposure terms were combined with outcome terms using AND operator to search for relevant literature. The exposure terms used included ADHD and all its variants such as hyperactive, diskinetic and attention deficit. The outcome related terms included injuries, accidents, burn*, fall*, traffic injuries; traffic accidents; transport accidents/injuries; fractures; lacerations; drowning; scalds and occupational/work related injuries. The design terms were controlled study; controlled clinical trial; case-control studies; cohort studies, and comparative studies.

**Data Extraction:**

Data were extracted by a two of the authors who were a psychology researcher and a psychiatry resident (Abdi & Nazari) and were also trained to do so by the study leaders (Amiri & Sadeghi) who supervised the process and conducted the quality check for the selected full text articles. 

**Statistical Analysis**

Meta-analysis was conducted through three different analysis plans. Plan 1 was conducted on all the comparative studies with acceptable quality. In plan 2 however, the cohort studies alone or together with case-control studies that were least likely of leading to biased pooled effect size due to reverse causality potential were analyzed. Odds ratios were pooled as the effect size both in plan 1 and plan 2. Standardized mean differences of ADHD rating scales were pooled as the overall effect size in plan 3 of the meta-analysis using the Galss method to calculate standardized mean. Most studies had defined ADHD as a dichotomous measure of having or lacking ADHD. The effect size of interest for these studies was quantified through the meta-analysis by odds ratios (OR) with 95% confidence intervals (plans 1& 2). Considering the high heterogeneity observed among the studies, the random effects model was applied to do the meta-analysis. Statistical heterogeneity was investigated through I2 statistic quantifying the amount of variation attributable to heterogeneity. I2 is calculated in a formula containing Cochrane’s heterogeneity statistic Q but with some advantages over Q. An I2>75% was considered to indicate high heterogeneity.17 As some studies had compared the mean difference of scores belonging to valid ADHD rating scales between the injured and uninjured subjects, a separate meta-analysis was done for these studies using standardized mean differences (plan 3).

A funnel plot was used to evaluate the presence of publication bias plotting study’s log OR as a function of its standard error. The asymmetry of funnel plot was tested assessed using Begg’s test of heterogeneity and graphical assessment. Statistical analysis was done using Stata 11 statistical package. 

## Results

**Figure 1:**
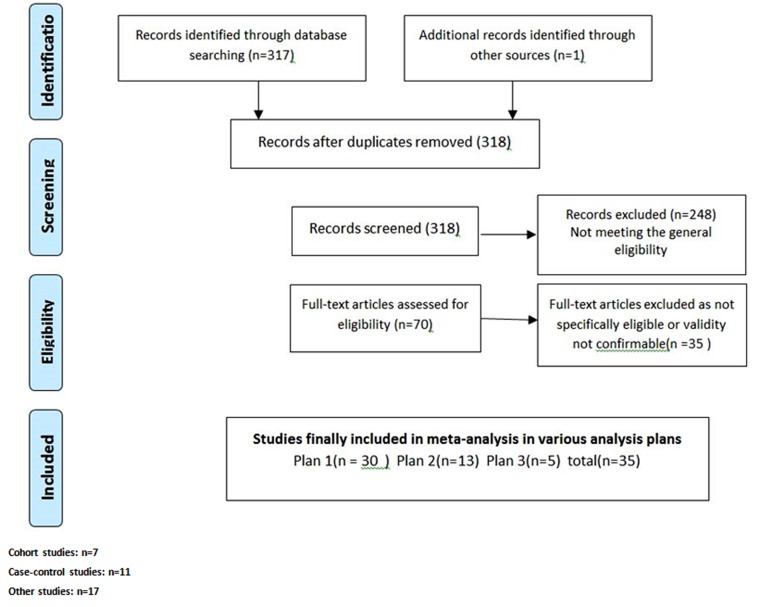
PRISMA flow chart for the studies systematically reviewed to investigate the association between attention deficit/hyperactivity disorder and injuries

**Figure 2:**
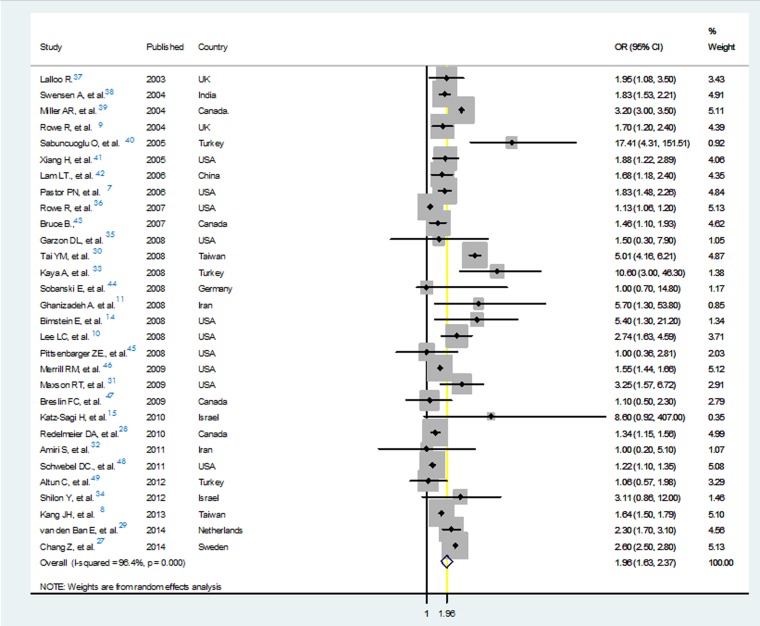
Forest plot of the meta-analysis of all comparative studies assessing the association of attention deficit/hyperactivity disorder and injuries

A total of 35 studies were finally selected for quantitative analysis (Fig.1). When including all the comparative studies with acceptable quality to be included for assessing the evidence on the association of ADHD diagnosis with injuries, such association was confirmed over the meta-analysis of 30 eligible studies. The pooled odds ratio was estimated to be 1.96(95% CI: 1.6-2.4) using random effects model in meta-analysis (Fig.2). Due to high amount of heterogeneity among the enrolled studies, random error models were used in all three analysis plans.

**Figure 3:**
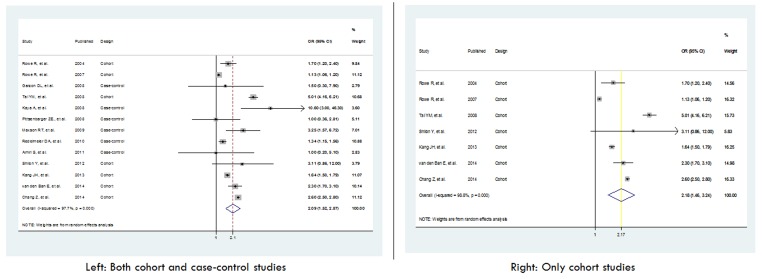
Forest plot of the meta-analysis of cohort and case-control studies assessing the association of attention deficit / hyperactivity disorder and injuries

As a conservative way of analysis through plan 2 of meta-analysis, only the cohort studies (7 studies) or cohort studies together with case-control studies(13 studies) which have assessed ADHD as a dichotomous variable were analyzed (plan 2 analysis). The pooled effect didn’t vary much from the full model yielding a pooled odds ratio of 2.1 and 2.17 respectively when both cohort and case-control studies or only cohort studies were included using random effects model (Fig.3).

Descriptions of the included case-control and cohort studies are given in Table 1.

**Table 1:** Summary of the characteristics of included case-control and cohort studies assessing the association between attention defi-cit/hyperactivity disorder(ADHD) and injuries

Study(Ref.)	Year published	Country	Study population	Age range	Gender	Sample size	Design	Exposure (ADHD definition)	Outcome (injury type)
(Chang, Lichtenstein, D'Onofrio, Sjolander, & Larsson, 2014)^27^	2014	Sweden	Those reigstered with ADHD diagnosis in Swedish national registers were followed up for one year to check for serious transport accidents documented in Swedish national registers.	18-46	60% males	17408	Cohort	ICD10 code F90	Serious transport accidents
(Redelmeier, Chan, & Lu, 2010)^28^	2010	Canada	Those hospitalized for road trauma(cases) or appendicitis(controls) in Ontario from 2002-2009	16-19	All males	3421	Case-control	Diagnosis based on medical records	Motor vehicle related trauma(codes v01-v99)
(Kang et al., 2013)^8^	2013	Taiwan	Those with ADHD diagnosis and controls without it followed for three years to check for injuries occurred.	4-12	78% males	3616 cases 18010 controls	Cohort	ICD9-CM diagnosis records for ADHD(codes 314 & 314.01) >=3 times	All injuries
(van den Ban et al., 2014)^29^	2014	Nether-lands	ADHD and control groups from PHARMO record linkage system (RLS) from 1985 on-wards, further linked to hospital admission records.	0-18	94% males	1289	Cohort	ADHD defined based on drug history of the child	All injuries
(Tai, Gau,& Gau, 2013)^30^	2008	Taiwan	Youthes with ADHD from Taiwan’s National Health Insurance Research Data-base (1997–2008) and age-sex matched controls.	6–18	50% Males(sex matched)	1965	Case-control	ICD9CM code 314.x	All injuries
(Rowe et al., 2004)^9^	2004	UK	Children from a nationally representative 10000 sample from British Child and Adolescent Mental Health Survey	5-15	NR	Ambiguity in numers.	Cohort	DSM-IV	Fractures selected
(Maxson, Lawson, Pop, Yuma-Guerrero, & Johnson, 2009)^31^	2009	USA	Patients aged 6 to 12 years, admitted to Children's Medical Center Dallas for specific injury mechanisms(cases) or appendicitis(controls)	6-12	67% males	133 cases and 157 controls	case–control study	Vanderbilt attention deficit/hyperactivity disorder parent rating scale(VADPRS)	Injury(all types)
(Amiri et al., 2011)^32^	2011	Iran	70 traumatic cases hospitalized due to traffic injuries and 70 age- sex- matched controls	18-61	98.6% males	140	case-control study	CAARS	Road traffic injuries
(Kaya et al., 2008)^33^	2008	Turkey	Patients with musculo-skeletal trauma treated as outpatients or admitted to hospital compared with controls with non-traumatic complaints	18-70	63% males 21	58 cases 30 controls	case-control study	DSM-IV-TR	Musculoskeletal trauma
(Shilon, Pollak, Aran, Shaked, & Gross-Tsur, 2012)^34^	2012	Israel	school-aged children with ADHD and their same-sex, similarly aged, non-ADHD-affected siblings	7-17	72% Males	29 cases 29 controls	Cohort	DSM-IV & Child Behav-ior Checklist, ADHD rating scale and development coordination disorder questionnaire	Accidental injuries
(Garzon, Huang, & Todd, 2008)^35^	2008	USA	children who presented to the emergency department comparing those with unintentional injuries with those who had non-injury-related dignosis	3-5	63% Males	47 cases and 46 controls	case–control study	Strengths and weaknesses of ADHD symptoms and normal behavior scale(SWAN)	All injuries
(Rowe, Simonoff, & Silberg, 2007)^36^	2007	USA	Twins in the community born 1974–1983 from Virginia twin study of adolescent behavioral development	8-17	50% Males(sex matched)	1st wave started with 2822 induviduals	Cohort	DSM-IIIR	Unintentional injuries during the past three months

**Figure 4:**
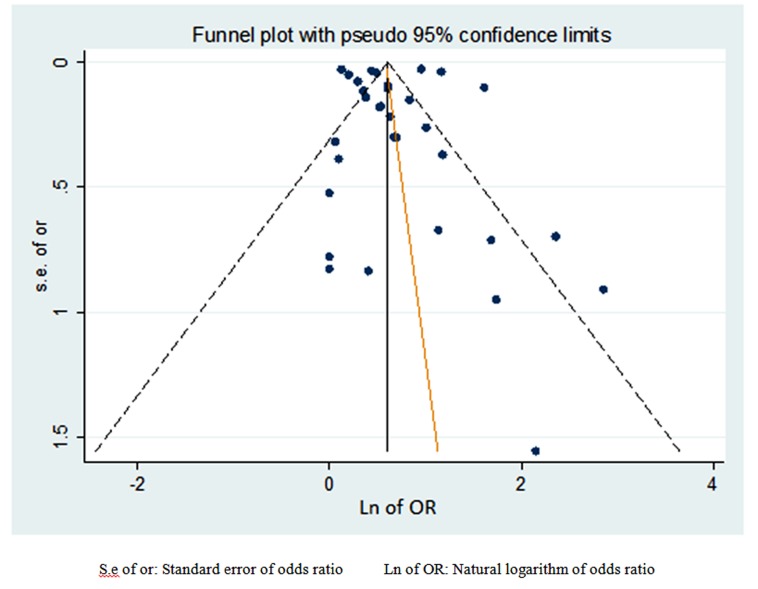
Funnel plot to assess the asymmetry due to heterogeneity or potential publication biasAsymmetry not confirmed using begg’s test on asymmetry of funnel plot

Begg’s test for exploring potential publication bias was not statistically significant with a borderline p-value and slight asymmetry was observed in graphical assessment of the funnel plot (Fig.4).

Most of the studies included in full meta-analysis model (30 studies) have investigated the association of ADHD with injuries regardless of the injury type, while others have focused on specific types of injuries such as fractures, burns, traffic injuries, motorcycle traffic injuries and dental injuries. A subgroup meta-analysis was done showing that the pooled odds ratio could be reduced by 1.8(1.45-2.3) when assessing the association of ADHD with injuries regardless of the injury type or injury mechanism.

**Figure 5:**
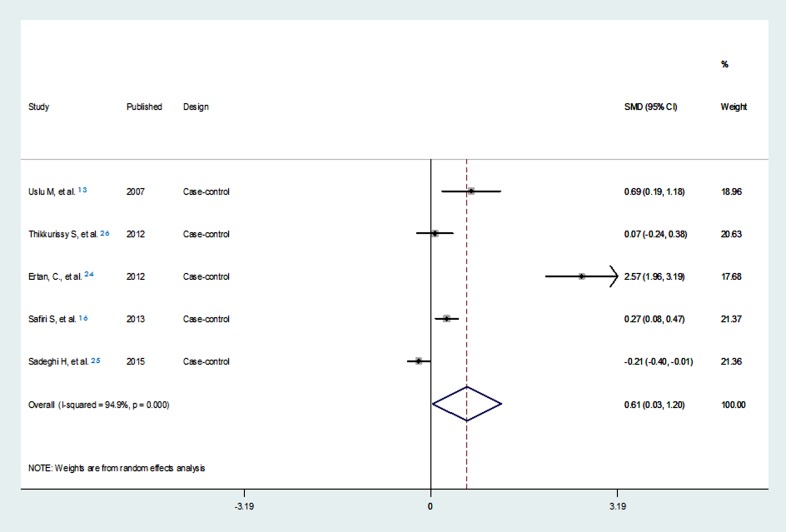
Forest plot of the meta-analysis of studies assessing the association of injuries and the score of attention deficit/hyperactivity disorder rating scales

Some studies have assessed the relationship between injuries and scores of ADHD rating scales such as CAARS or other rating scales and have reported the difference in these screening scores between the injury victims and non-injured controls. The mean standardized effect sizes were found to be nearly high (Pooled SMD=0.61) through the meta-analysis , but the validity of model results couldn’t be well accepted due to data limitations such as small number of studies and potential publication bias (Fig.5).

**Figure 6:**
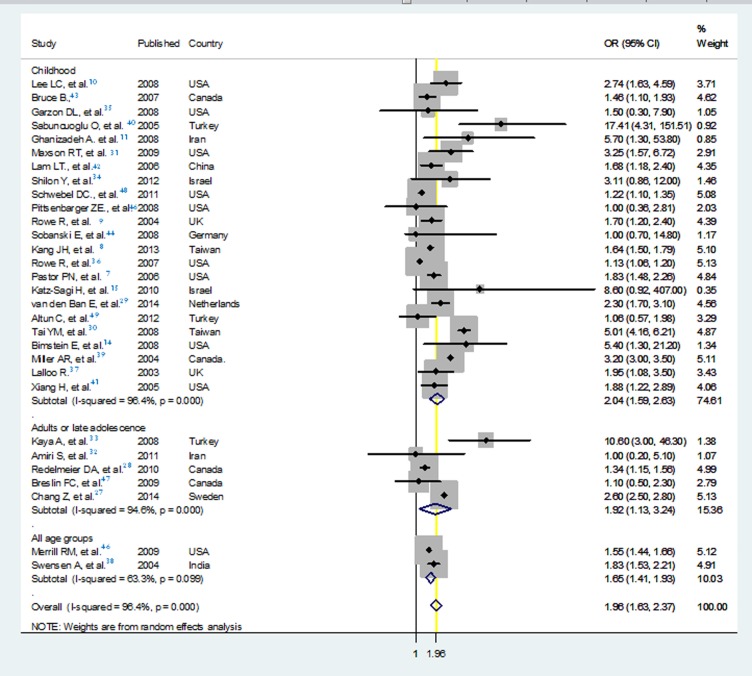
Forest plot of the meta-analysis of all comparative studies assessing the association of attention deficit/hyperactivity disorder and injuries according to age groups

To compare the age groups with respect to the potential association of ADHD and injuries, a subgroup meta-analysis was run showing that studies restricted to children had the highest pooled odds ratio equal to 2.04 (Fig.6).

## Discussion

The present systematic review revealed a strong association between ADHD and injuries. Some characteristics of patients with ADHD could reasonably explain such association. First of all, it could be the risk-taking behaviors that are much more prominent in ADHD. Risk-taking, a well-known predictor in road traffic injuries, could easily increase the risks of other types of injuries such as either falls or burns. Psychological explanations for risky behavior among young people have been discussed from various perspectives, including the psychodynamic approach, the cognitive approach, and the character and human motivation approach. Secondly, it is the loss of concentration which seems to have the most prominent role on traffic and occupational as well as other types of injuries. Moreover, it seems quite easy for a talkative driver to lose concentration on driving when he/she finds a co-traveler eager to listen. Actually the three factors as errors, lapses and violations are the points of importance when investigating the risks of driving among ADHD patients. Simulation studies have shown that the driving performance of ADHD patients may be improved regarding these elements after pharmacologic treatment; however, there is not a solid evidence on the efficacy of cognitive, behavioral or educational programs in reducing the driving risks of patients with ADHD. ^18^ The hyperactivity part of ADHD although not plausible to have a direct effect on driving risks seems to be of importance in childhood burns. Considering that it is a common risk in some developing countries where children are overcrowded in small living areas and some traditional heaters with a boiling kettle or pot on them are usually set in the center of living room, hyperactivity shows its plausible role to increase the risk of getting burned children with a higher chance of running around frequently. No doubt the situation gets more risky when attention deficit is added to the scenario.^11,19-21^

This systematic review was interested in investigating the association of ADHD and injuries and looking for the evidence whether ADHD can affect the occurrence of injuries. The association between ADHD and injuries could be ambi-directional in case of traumatic brain injuries. It has been suggested that traumatic brain injury may also cause ADHD and this should be carefully addressed while investigating the association of ADHD and injuries.^22,23^ To avoid the reverse causality problem in introducing bias in assessment of the effect of ADHD, we excluded the studies specifically conducted on the association of ADHD and traumatic brain injuries. However, as other studies might also have included brain traumas without a reference to that, we conducted a secondary meta-analysis restricting the studies only to cohort and case control studies at analysis plan 2 in which the direction of association could be interpreted and this confirmed the overall finding of the meta-analysis as in plan 1 conducted on all included studies. The detection of the cause-effect temporality direction is quite straightforward in cohort studies and all the cohort studies in this review supported the evidence on effect of ADHD. In classic case-control studies, the detection of the direction of associations could be biased when the outcome of interest is a disease with long variant latency or incubation period and/or the exposure is a transient hard to retrospectively measure condition. However, for assessing the association of ADHD and injuries, this is of little concern due to the fact that injuries are sudden outcomes without a long latency prior to the event and ADHD is a chronic nearly stable exposure existing from childhood. If the symptoms of ADHD are assessed soon after an injury occurs with reference to the time before the injury incidence, the detection of the direction of ADHD and injury events will not be a problem. Nevertheless, selection bias and information bias are always source of concern in case-control studies.

The way ADHD is assessed is another issue to be discussed. Few of the studies on association of ADHD and injuries were case-control studies which assessed the ADHD status through rating scales such as Conner’s scales.^13,16,24-26^ Regardless of the variety in rating scales used by different studies, the point in such assessment is that these studies just assess the association of the ADHD rating score with injuries rather than association of ADHD and injuries unless a cutoff point is used to dichotomize the score or the patients with higher scores are later assessed for the diagnosis of ADHD through clinical interviews done by a psychiatrist. However, the research question may also be different whether people with higher scores, regardless of their final diagnosis, are at higher risk of injuries or not. The use of a numeric scale in this manner may even affect the statistical power.

Although not in line with the research question in this study, we found through the methodological assessment of articles that some studies published as case-control studies or stated to be case-control studies had a cross-sectional design. The authors of current review categorized these studies according to the design distinguished through the methodological assessment by an epidemiologist rather than the terminology used by the authors of the original studies.

## Conclusion

Those with ADHD are nearly two times more likely to be injured. Children, adolescents and adults with ADHD are all at higher risks of various types of mostly unintentional injuries. Clinicians and health care providers who are in contact with people having ADHD should inform them about increased risk of injuries. Health policy makers should consider public management of ADHD in order to decrease risk of injuries at the community. Improving public knowledge on symptoms, consequences, and the management of ADHD is strongly recommended.
